# Heterogeneity in the associations between common mental disorders and labour outcomes – a population study from southern Sweden

**DOI:** 10.1186/s12889-020-09348-3

**Published:** 2020-08-26

**Authors:** Johan Jarl, Anna Linder, Hillevi Busch, Anja Nyberg, Ulf-G Gerdtham

**Affiliations:** 1 Health Economics Unit, Department of Clinical Sciences Malmö, Lund University, Box 117, 221 00 Lund, Sweden; 2 Centre for Economic Demography, Lund University, Lund, Sweden; 3 Public Health Agency of Sweden, Stockholm, Sweden; 4 Department of Healthcare Governance, Region Skåne, Sweden; 5 Department of Economics, Lund University, Lund, Sweden

**Keywords:** Mental disorders, Labour outcomes, Inequality, Sweden

## Abstract

**Background:**

Previous research has shown that Common Mental Disorders (CMD) are unequally distributed between population subgroups, but we know less about how labour outcomes following such disorders are distributed. Our aim is to investigate how the labour outcomes following a CMD diagnosis differ over sex, age, schooling and country of birth.

**Methods:**

We use a population sample from southern Sweden of patients diagnosed with CMD during calendar years 2009–2011, and a matched general population control group, to study labour market outcomes three years following diagnosis. Logistic regression is used to study the associations between a CMD diagnosis and outcomes in employment, sick leave, and disability pension. Interaction analysis is used to study heterogeneity in these associations.

**Results:**

CMD diagnosis is associated with reduced employment and increased odds of sick leave and disability pension. Following a CMD diagnosis, men and higher educated individuals have higher odds of non-employment and sick leave compared to women and the lower educated. Foreign-born individuals have higher odds of non-employment and lower odds of sick leave, compared to individuals born in Sweden. Heterogeneity appears to be present also based on age. Younger age is associated with higher odds of non-employment and disability pension and lower odds of sick leave, following a CMD diagnosis.

**Conclusions:**

Heterogeneity in labour outcomes following a CMD diagnosis sometimes contributes to and sometimes mitigates inequalities in employment, sick leave and disability pension between population subgroups. When developing new strategies to tackle mental ill-health in the population, it may therefore be motivated to consider not only inequalities in the prevalence of mental disorders but also heterogeneity in associated adverse labour outcomes.

## Background

All groups in the population are at risk of mental disorders irrespective of sex, age, ethnicity and socioeconomic status (SES), but the prevalence is not equally distributed among all population subgroups. Educational and income gradients in mental health are well established in the literature [1] and women are in general more exposed to common internalizing disorders such as depression and anxiety than men [2]. Migration status and ethnicity have been found both positively [3] and negatively [4] associated with mental disorders, but these relationships are likely to vary depending on the specific context and background characteristics. Prevalence of mental disorders also vary over the lifecycle depending on the health problem under consideration, but three-quarters of all lifetime mental disorders onset before the mid-20s [5].

Poor mental health is moreover linked to adverse social and economic living conditions. Child and adolescent mental health problems affect human capital accumulation [6–10] and future labour opportunities [9, 11–15]. Among adults, individuals who suffer from mental health problems are more likely to be unemployed or outside the labour market [16–25] and to be absent from work [16, 19, 23]. The labour consequences have in some studies been found to be worse among men [16, 17, 20, 23, 25], and in some worse among women [21, 24]. In a recent literature review it was found that sickness absence due to anxiety and depression is similar between women and men but that men are more severely affected in terms of disability pension [26]. Sickness absence also appears to differ with SES [26]. In general though, little attention is paid in existing research to differences in the association between mental disorders and labour outcomes that are related to, for example, sex, age, SES and migration status. This is an important research gap since if such heterogeneity exists, it could increase inequalities in wellbeing both due to inequalities in labour outcomes and inequalities in health. With the current study, we try to mitigate this research gap. Our purpose is to investigate how the labour outcomes following a diagnosis for Common Mental Disorders (CMD) differ over sex, age, schooling and country of birth.

## Method

For the purpose of our study, we employ a dataset comprising of all individuals who received a diagnosis of a CMD in outpatient care in Region Skåne, Sweden during the years 2009–2011. We chose to focus on CMD as this has been a prioritised policy area in order to reduce sickness absence rates in Sweden and we use the same definition [27]: (ICD-10 code) depressive episode (F32), recurrent depressive episode (F33), phobic anxiety disorders (F40), panic disorders (F410), generalised anxiety disorder (F411), unspecified anxiety disorder (F419), obsessive-compulsive disorder (F42), post-traumatic stress disorder (F431), other reactions to severe stress (F438), unspecified reaction to severe stress (F439). Individuals who had been hospitalized, i.e. received an inpatient diagnosis, due to these disorders are excluded, as these cases are not considered CMD. The sample is limited to ages 20–59 years at the time of diagnosis to make sure that no one reaches retirement age (normally 65 years of age) during the follow-up (*n* = 41,716). Follow-up is three years including the year of diagnosis.

A control group was matched to the cases from a general population sample (Living Conditions Surveys – ULF/SILC). In order to estimate the total effect of CMD, a healthy control group was created by excluding individuals who had received an inpatient or outpatient CMD diagnosis during follow-up according to national patient registers (National Board of Health and Welfare). A nearest neighbour 1:1 propensity score matching approach without replacement and a maximum calliper of 0.001 was applied. The propensity score was based on sex, age, schooling, and whether the person was Swedish- or foreign-born. Sex is divided into male and female and schooling is categorized following the Swedish schooling system (mandatory education ≤9 years, secondary education 10–12 years, and higher education ≥13 years). We match on age continuous in years but in the regression analyses age is categorized into four groups, 20–29, 30–39, 40–49, and 50–59 years, respectively. Foreign-born captures factors associated with not having a Swedish background, e.g. language barriers, cultural perceptions, and discriminations, as this might be a source of heterogeneity in the current context. More detailed information was unavailable for the current study. Out of the 41,716 cases (individuals with a CMD diagnosis between the year 2009–2011), 38,304 were matched to a control individual. Unmatched individuals (*n* = 3412; 8.2%) were dropped from the analysis, leaving a final sample of 76,608 individuals (dropped cases were on average more likely to be women, younger, foreign born, and have low education). Baseline year for the controls was set to the year of diagnosis for each matched case, respectively.

Yearly information on labour outcomes (employment, sick leave and disability pension), as well as several sociodemographic characteristics, were added for the follow-up years as well as the year before from the Longitudinal Integrated Database for Health Insurance and Labour Market Studies (LISA) [28]. Employment follows the definition used by Statistics Sweden and is determined by employment during the month of November each year. To facilitate interpretation, the inverse of employment is used in the regression models; non-employment is equal to 1 if not employed in November at least one of the three follow-up years, and 0 otherwise. Sick leave is also defined as a binary variable and equal to 1 if at least one case of sickness absence (all causes) longer than 14 days during the follow-up, and 0 otherwise. It is only after 14 days that a sick leave is reported to the Swedish Social Insurance Agency, therefore shorter cases of sickness absence are not included in the registers. In Sweden, sickness- and activity compensation (from now on referred to as “disability pension”) is granted for lasting reduced work capacity and is granted on full- or part-time reflecting the degree of reduced work capacity. It is not necessarily a permanent state and the individual’s work capacity is regularly re-evaluated with the goal that the recipient should return to work. An individual is defined as on disability pension if they at any time during the follow-up hold disability pension (all causes, full or part-time), and 0 otherwise. The labour outcomes are not mutually exclusive, an individual can, in theory, be defined as employed, on sick leave and on disability pension at the same time. It is also possible to be on sick leave while not employed (covered by the national social insurance. Out of the 7749 individuals that hold disability pension in the year before diagnosis in our study sample, more than one-third were also in employment during that year, indicating that part-time disability pension is quite common. Moreover, during the follow-up around one-sixth of these individuals do not hold any disability-pension, indicating that holding disability pension is not a permanent state. Still, it is likely that disability pension is linked to both CMD and the other labour outcomes, and that disability pension the year before diagnosis has a strong prediction for later disability pension. Therefore, the labour outcomes following CMD diagnosis are analysed in the full sample in the main analysis, but we perform additional test to investigate the results sensitivity by excluding individuals with disability pension the year before diagnosis.

### Statistical analyses

Estimating the relationship between mental health and labour outcomes is complicated by several issues. The first issue is related to two-way causality – it is likely that the causal relationships between mental health and labour outcomes go both ways. The second issue is related to unobserved heterogeneity – it is possible that individuals who suffer from mental disorders differ from healthy individuals in unobserved ways which cannot be controlled for. Due to these issues, it is complicated to draw causal inferences from observed differences in the studied labour outcomes between individuals with and without CMDs. In this study, we use propensity score matching to create a control group and estimate the relationship between CMDs and subsequent labour outcomes. A key identifying assumption when using matching is to match on all observable covariates that are important both for being diagnosed with a CMD and for the labour outcomes under study. The idea is that if cases and controls differ less in terms of these covariates (e.g. schooling), they might also differ less in terms of relevant unobserved covariates (e.g. preferences). Thus, by matching cases to controls based on sex, age, schooling and whether Swedish- or foreign-born, we do not only adjust for the matching variables, but also to some extent unobserved variables correlated to the observed variables. Moreover, since we want to study the labour outcomes following a CMD diagnosis we control for the outcomes one year before diagnosis. However, since there is no way of testing to what extent we have adjusted for relevant unobserved heterogeneity we take a conservative approach and consider our results as reduced bias estimates and are careful in making strict causal interpretations.

Labour outcomes the year before diagnosis and over the follow-up period are shown graphically for the case group, in total and separately for men and women, by age-group, schooling and country of birth. Logistic regression analyses are performed to study the association between a CMD diagnosis and subsequent labour outcomes. Interaction effects are included to show the difference in the strength of this association based on sex, age, schooling and country of birth. The interaction effect, for example between diagnosis and sex, shows the difference in associations between diagnosis and the labour outcome for men compared women, as a ratio of odds ratios. A statistically significant interaction effect between diagnosis and sex indicate that there is significant heterogeneity in the association between diagnosis and the labour outcome based on sex.

The interaction effect is difficult to interpret in terms of effect size. We therefore, based on the logistic regressions, estimate the predicted probabilities of each labour outcome in the case and control groups. The predicted probabilities are the average of each individual’s predicted probability for the different labour outcomes, i.e. based on individual characteristics. These give an indication in absolute terms of how the labour outcomes differ over sex, age, schooling and country of birth. Additionally, these predicted probabilities give an indication of how the potentially heterogeneous labour outcomes could affect the current distribution of employment, sick leave and disability pension.

Statistical significance is considered at the 5% level and all analyses are conducted in Stata 14. Ethical approval has been obtained from Lund Ethical Review Board (2015/204).

## Results

The study population is described in Table 1. Given the strict limit on the maximum distance between the case and control groups in the propensity score matching, the distributions of these variables are close to identical, even though statistical significant differences were noted due to the large study sample. Differences between the case and control groups regarding area of residence are due to the design of the data material; cases are from Skåne while controls are from the whole of Sweden. There are significant differences between the case and control groups in terms of labour outcomes both in the year before diagnosis/inclusion in the study (hereafter before diagnosis), and during the follow-up period. The cases are to a higher extent non-employed, on sick leave and hold disability pension. Income is lower in the case group compared to the control group.

**Table 1:** Descriptive statistics of study population

	Case (%) (*n* = 38,304)	Control (%) (n = 38,304)	Total (%) (*n* = 76,608)
Sex			
Female	63	63	63
Male	37	37	37
Age ***			
20–29	18	18	18
30–39	27	26	26
40–49	30	30	30
50–59	26	26	26
Education ***			
Mandatory	14	14	14
Secondary	48	49	48
Higher	37	38	37
Foreign born	18	19	19
Resident of Skåne ***	100	13	56
Year of inclusion in the study			
2009	26	26	26
2010	40	40	40
2011	33	33	33
Employed year −1 ***	79	83	81
On sick leave year −1 ***	19	9	14
Sick leave (days) year − 1 ***	20.79 (0.35)	6.00 (0.17)	13.33 (0.19)
Disability pension year −1 ***	14	6	10
Disposable income year −1 (in quintiles) ***			
Income Q1	22	18	20
Income Q2	21	19	20
Income Q3	20	20	20
Income Q4	19	21	20
Income Q5	17	23	20
Not employed year 1–3 ***	40	21	31
On sick leave year 1–3 ***	43	20	32
Disability pension year 1–3 ***	12	6	9

Note: Differences between cases and controls are studied in univariable analyses using t-test and Chi2-test. For continuous variables standard errors are presented in parenthesis. Year −1 corresponds to the year before diagnosis. Year 1 is the year of diagnosis and years 2 and 3 are the two years following year of diagnosis. Statistically significant differences are noted on *** 1% level

**Fig. 1:**
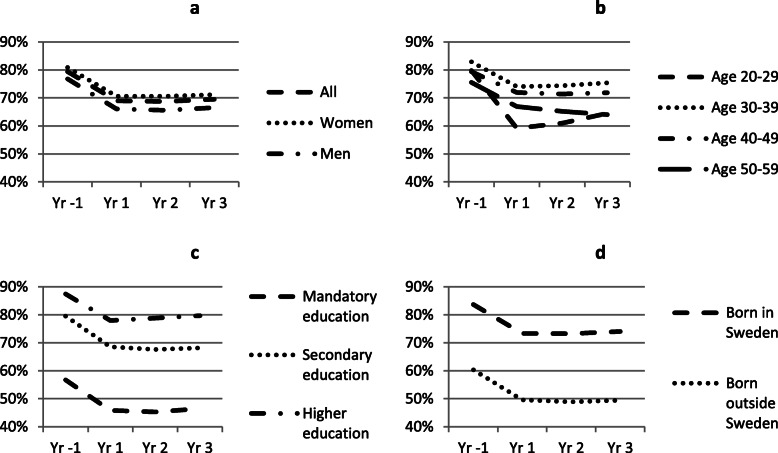
Proportion in employment before and after CMD diagnosis. Note: Yr − 1 corresponds to the year before CMD diagnosis, Yr 1 is the year of diagnosis and Yr 2 and 3 are the two years following year of diagnosis

**Fig. 2:**
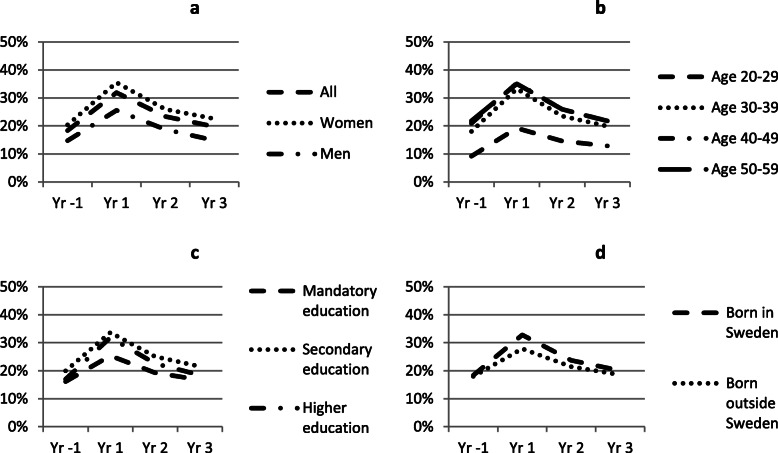
Proportion on sick leave before and after CMD diagnosis. Note: See note Fig. 1. In 2b the trend for 40–49-year-olds almost perfectly coincides with the trend for 50–59-year-olds

**Fig. 3:**
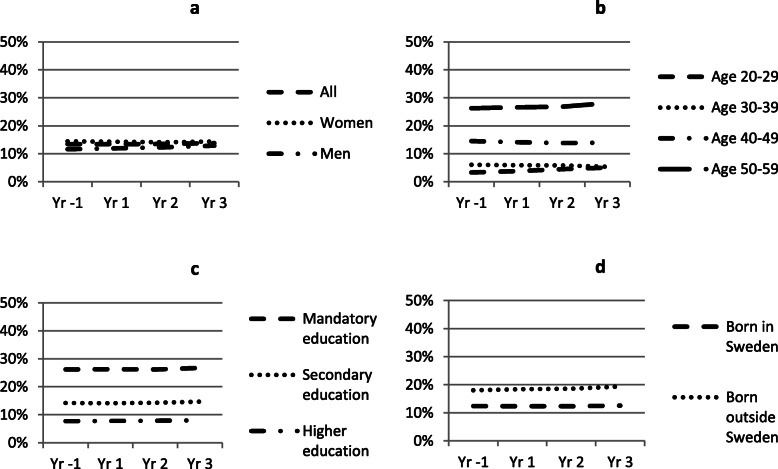
Proportion on disability pension before and after CMD diagnosis. Note: See note Fig. 1

In Figs. 1, 2, and 3 we show trends in employment, sick leave and disability pension for all cases, one year before and up to three years after a CMD diagnosis. Separate trends are shown by sex (a), age (b), education (c) and country of birth (d). Employment rate declines in all subgroups the year of diagnosis (Fig. 1a–d), especially among the youngest age group, but this group also show a relatively faster recovery. In the oldest age group employment rate continue to fall during the follow-up years. Trends in sick leave are similar in all groups, increase substantially in the year of diagnosis and decline thereafter (Fig. 2a–d). However, sick leave appears to increase relatively less among the lowest educated and among those born outside of Sweden. Trends in disability pension are similar based on sex, education and country of birth, but appear to differ somewhat with age where disability pension increase more among the youngest and oldest age groups, compared to the middle-aged groups (Fig. 3a–d).

### Employment

A CMD diagnosis is associated with increased odds of non-employment as shown by the estimated odds ratio of 3.74 [3.58–3.91], see Table 2 column 1. The interaction effects show that given a CMD diagnosis, employment status differ significantly based on sex, schooling, age and country of birth (see column 2). The odds of non-employment are significantly higher among men compared to women 1.30 [1.19–1.43] as a ratio of the odds ratio. The odds ratio for men was (3.92*1.30 =) 5.96 and 3.92 for women. The odds of not being employed after a CMD diagnosis are moreover significantly higher among those highest educated compared to those with mandatory education 1.15 [1.00–1.31], among those born outside of Sweden compared to those born in Sweden 1.18 [1.05–1.31], and significantly lower among all the older groups compared to the youngest group, among 30–39-year olds 0.85 [0.74–0.96], among 40–49-year olds 0.85 [0.75–0.96], and among 50–59 years 0.71 [0.63–0.81].

**Table 2:** Logistic regression (odds ratios) – The association between CMD and subsequent labour outcomes

	Non-employed Model 1 (1)	Non-employed Model 2 (2)	Sick leave Model 1 (3)	Sick leave Model 2 (4)	Disability pension Model 1 (5)	Disability pension Model 2 (6)
No CMD	Ref.	Ref.	Ref.	Ref.	Ref.	Ref.
CMD	3.74 [3.58–3.91]	3.92 [3.38–4.54]	2.84 [2.74–2.94]	1.79 [1.57–2.04]	1.31 [1.16–1.47]	2.20 [1.37–3.52]
Outcome year −1	34.15 [32.06–36.38]	34.61 [32.48–36.88]	5.74 [5.48–6.02]	5.73 [5.46–6.00]	928 [809–1066]	926 [807–1063]
Man*CMD		1.30 [1.19–1.43]		1.26 [1.17–1.36]		1.19 [0.93–1.54]
Secondary education*CMD		0.92 [0.82–1.05]		1.40 [1.26–1.55]		0.99 [0.75–1.30]
Higher education*CMD		1.15 [1.00–1.31]		1.76 [1.58–1.97]		1.15 [0.82–1.62]
30–39 years*CMD		0.85 [0.74–0.96]		1.07 [0.96–1.20]		0.56 [0.33–0.93]
40–49 years*CMD		0.85 [0.75–0.96]		1.13 [1.01–1.26]		0.51 [0.32–0.81]
50–59 years*CMD		0.71 [0.63–0.81]		1.00 [0.90–1.12]		0.59 [0.37–0.94]
Foreign born*CMD		1.18 [1.05–1.31]		0.79 [0.72–0.87]		0.83 [0.63–1.09]
n	76,608	76,608	76,608	76,608	76,608	76,608
Pseudo-R^2^	0.39	0.39	0.14	0.14	0.78	0.78

Note: Non-employment in November at least one of the three follow-up years. Sick leave > 14 days and disability pension at least once during the follow-up. The interaction effect (denoted by *) shows the difference in associations between CMD diagnosis and the labour outcome for the interacted group compared to the reference group, as a ratio of odds ratios. The reference groups are women, mandatory education, 20–29 years of age and born in Sweden. We control for sex, education, age-group, being foreign-born, income and year of diagnosis/inclusion in the study in all regressions. Income is measured by disposable income in the year before diagnosis and is stratified in quintiles

The estimated predicted probabilities show the magnitude of the difference in absolute terms over subgroups, see Table 3. For example, predicted non-employment during at least one of the follow-up years is similar between men and women without diagnosis (23%) but higher among men with diagnosis (41%) compared to women with diagnosis (37%). Moreover, predicted non-employment differ substantially based on schooling, age and country of birth. Higher education predicts lower non-employment among those with and without diagnosis (35 and 20%, respectively) compared to those with mandatory schooling (46 and 27%, respectively) and secondary schooling (39 and 24%, respectively), but the difference in employment due to diagnosis is relatively larger among the highest educated. These results also show that a CMD diagnosis predicts relatively high levels of non-employment in some of the subgroups, among the lowest educated (46%), among the youngest age group (50%) and among those who are foreign-born (46%).

**Table 3:** Predicted probability of non-employment, sick leave and disability pension during the three follow-up years

	Non-employed No CMD [1]	Non-employed CMD [2]	Sick leave No CMD [3]	Sick leave CMD [4]	Disability pension No CMD [5]	Disability pension CMD [6]
Women	0.23 [0.23–0.24]	0.37 [0.37–0.38]	0.26 [0.25–0.26]	0.45 [0.45–0.46]	0.08 [0.08–0.09]	0.09 [0.09–0.09]
Men	0.23 [0.22–0.24]	0.41 [0.40–0.41]	0.15 [0.15–0.16]	0.34 [0.34–0.35]	0.09 [0.08–0.09]	0.09 [0.09–0.10]
Mandatory education	0.27 [0.26–0.28]	0.46 [0.45–0.47]	0.24 [0.23–0.25]	0.36 [0.35–0.37]	0.09 [0.09–0.09]	0.09 [0.09–0.10]
Secondary education	0.24 [0.24–0.24]	0.39 [0.39–0.4]	0.24 [0.24–0.25]	0.43 [0.43–0.44]	0.09 [0.08–0.09]	0.09 [0.09–0.09]
Higher education	0.20 [0.19–0.20]	0.35 [0.34–0.36]	0.19 [0.18–0.19]	0.40 [0.40–0.41]	0.08 [0.07–0.08]	0.09 [0.08–0.09]
20–29 years	0.28 [0.27–0.29]	0.50 [0.49–0.52]	0.17 [0.16–0.18]	0.32 [0.31–0.33]	0.07 [0.06–0.08]	0.08 [0.08–0.09]
30–39 years	0.21 [0.21–0.22]	0.36 [0.35–0.36]	0.23 [0.23–0.24]	0.44 [0.43–0.45]	0.07 [0.06–0.07]	0.07 [0.07–0.08]
40–49 years	0.20 [0.19–0.21]	0.34 [0.33–0.34]	0.23 [0.22–0.23]	0.44 [0.43–0.45]	0.08 [0.08–0.08]	0.08 [0.08–0.09]
50–59 years	0.25 [0.24–0.26]	0.39 [0.38–0.4]	0.23 [0.22–0.24]	0.42 [0.41–0.43]	0.10 [0.09–0.10]	0.10 [0.10–0.10]
Born in Sweden	0.22 [0.22–0.23]	0.37 [0.37–0.37]	0.22 [0.22–0.22]	0.42 [0.42–0.43]	0.08 [0.08–0.09]	0.09 [0.09–0.09]
Foreign born	0.26 [0.25–0.27]	0.46 [0.45–0.47]	0.22 [0.21–0.23]	0.37 [0.36–0.39]	0.09 [0.08–0.09]	0.09 [0.09–0.09]

Note: We control for sex, education, age, being foreign-born, income, and the outcome variable the year before diagnosis in all regressions. Income is measured by disposable income in the year before diagnosis and is stratified in quintiles. See Supplementary file 1 for the full regression results. Predicted probabilities of outcomes are the average over individuals calculated as observed for other variables

### Sick leave

CMD diagnosis is associated with increased odds of sick leave by 2.84 [2.74–2.94]. The interaction effects show that given a CMD diagnosis, sick leave differ significantly between men and women where the odds (again as a ratio of ratios) are higher among men 1.26 [1.17–1.36]. Schooling is also a significant factor and sick leave is significantly higher among those with secondary education 1.40 [1.26–1.55] and higher education 1.76 [1.58–1.97], compared to those with only mandatory education. The age-stratified analyses show that sick leave is significantly higher only among 40–49-year olds where a CMD diagnosis is associated with 1.13 [1.01–1.26] higher odds compared to the reference group 20–29-year olds. Sick leave after a CMD diagnosis is moreover significantly lower among those born outside of Sweden compared to those born in Sweden 0.79 [0.72–0.87].

The predicted probability of sick leave is lower among men compared to women, 34 and 15% for men with and without diagnosis respectively compared to 45 and 26% for women with and without diagnosis respectively. Moreover, schooling predicts lower sick leave among those without a CMD diagnosis (19% among the highest educated compared to 24% in the secondary and mandatory educated groups) but higher sick leave rates among those with a CMD diagnosis (40 and 43% among the higher and secondary educated groups respectively, compared to 36% in the mandatory educated group). Predicted sick leave does not differ much between the middle-aged and the oldest age groups, but in the youngest group predicted sick leave is substantially lower compared to the older groups. Predicted sick leave is 17% among 20–29-year olds without diagnosis compared to 23% among older groups without diagnosis, and 32% among 20–29-year olds with a diagnosis compared to 42–44% among older groups with a diagnosis. Country of birth appears to matter for sick leave only among individuals with a diagnosis where predicted sick leave is 42% for those born in Sweden and 37% among those foreign-born. Predicted sick leave does not differ based on country of birth among those without a CMD diagnosis. Similarly as for employment, these results show that a CMD diagnosis predicts relatively high risks of sick leave in some of the subgroups, especially among women.

### Disability pension

For the last labour outcome, we find that CMD diagnosis is associated with increased odds of holding disability pension by 1.31 [1.16–1.47]. The interaction effects show that given a CMD diagnosis, disability pension differ significantly only by age. The odds of holding disability pension during the three years following a CMD diagnosis are lower among 30–39-year olds 0.56 [0.33–0.93], among 40–49-year-olds 0.51 [0.32–0.81] and among 50–59-year olds 0.59 [0.37–0.94], compared to the youngest age group 20–29-year-olds. The association between CMD diagnosis and disability pension does not differ significantly between men and women, or based on education and country of birth.

The predicted probability of holding disability pension does not vary by sex, schooling or country of birth among those with a CMD diagnosis. For individuals without a diagnosis, predicted disability pension is only marginally lower among women, the higher educated and among those born in Sweden. For age, however, predicted disability pension differs somewhat more. Among individuals without a CMD diagnosis, predicted disability pension increases with age from 7% among 20–29- and 30–39-year-olds to 8 and 10% among 40–49- and 50–59-year-olds, respectively. Among individuals with a CMD diagnosis, predicted disability pension is 8% among 20–29- and 40–49-year-olds and 7 and 10% among 30–39- and 50–59-year-olds, respectively.

### Sensitivity analyses

Holding disability pension the year before diagnosis is a strong predictor of disability pension during follow-up, see Table 2 column 5. Disability pension is likely a predictor of CMD, and perhaps also predict non-employment and sick leave. Therefore, we perform our analyses (logistic regression with interaction effects on the association between CMD diagnosis and the three labour market outcomes) on a sample where individuals that held disability pension in the year before diagnosis are excluded. This has no meaningful impact on the interpretation of the results on non-employment or sick leave, however, the main results for disability pension changes somewhat, see Table 4. A CMD diagnosis is associated with substantially higher odds for holding disability pension (11.45 [2.44–53.81]) but the age-related heterogeneity that was found in the main analyses seems less important. The interaction effects show that the odds of disability pension after a CMD diagnosis are now significantly lower only among 30–39-year olds compared to 20–29-year-olds. Moreover, excluding those who held disability pension the year before diagnosis reveals heterogeneity based on country of birth, foreign-born individuals now have significantly higher odds of holding disability pension after a CMD diagnosis compared to those born in Sweden, 3.96 [1.35–11.66].

**Table 4:** Sensitivity analysis: Logistic regression (odds ratios) – The association between CMD and subsequent disability pension

	Disability pension DP Yr − 1 excluded [1]
No CMD	Ref.
CMD	11.45
	[2.44–53.81]
Man*CMD	1.44
	[0.69–3.00]
Secondary education*CMD	1.67
	[0.73–3.80]
Higher education*CMD	1.94
	[0.74–5.10]
30–39 years*CMD	0.13
	[0.02–0.66]
40–49 years*CMD	0.25
	[0.05–1.21]
50–59 years*CMD	0.26
	[0.06–1.16]
Foreign born*CMD	3.96
	[1.35–11.66]
n	68,861
Pseudo-R^2^	0.14

Note: Disability pension at least once during the three follow-up years. The interaction effect (denoted by *) shows the difference in associations between CMD diagnosis and the labour outcome for the interacted group compared to the reference group, as a ratio of odds ratios. The reference groups are women, mandatory education, 20–29 years of age and born in Sweden. We control for sex, education, age-group, being foreign-born, income and year of diagnosis/inclusion in the study. Income is measured by disposable income in the year before diagnosis and is stratified in quintiles

## Discussion

In this study, we investigate the association between CMD diagnosis and labour outcomes using a population sample on all individuals diagnosed with CMD in Region Skåne during the years 2009–2011. CMD diagnosis is associated with adverse labour outcomes, but more importantly, these associations differ based on sex, schooling, age, and country of birth.

Both men and women who suffer from CMD are at increased risk of being non-employed, on all-cause sick leave and hold all-cause disability pension. However, men appear to have worse outcomes than women in employment and sick leave. Previous research has shown that the association between CMD and sick leave is similar between men and women [29–31] while men with mental disorders more often are found in disability pension [32–34]. For the relationship between CMD and employment, both men [16, 17, 20, 23, 25] and women with mental disorders [21, 24] have been found to be disadvantaged. In a Swedish study, it was found that women with depressive symptoms were at higher risk of losing their job than men with depressive symptoms [35]. Still, an overall assessment of the previous literature implies that men with CMD have poorer labour outcomes compared to women with CMD, which is in line with the results of this study. It has been suggested that men, at the same level of morbidity as women, have a lower tendency to report mental health problems [36, 37]. Consequently, mental disorders among men may reflect more severe symptomatology than among women at the time of diagnosis, which could explain the stronger association between CMD diagnosis and adverse labour outcomes found among men.

Our results show that CMD diagnosis is associated with adverse labour outcomes among all education levels. An educational gradient is also revealed. Surprisingly, and in contradiction to earlier associative evidence that has linked low SES to higher depression- and anxiety-related sickness absence [38, 39], we find that schooling increase the odds of non-employment and sick leave, following a CMD diagnosis. In general, low SES groups have fewer resources to invest in treatment, less social support and greater insecurities in employment, factors that could explain higher non-employment and sick leave among those with low SES, but there are potential explanations for our findings as well. High education has been shown to be positively associated with work-related mental health problems [40]. High educated individuals may have jobs related to more responsibilities and stress, which in combination with CMD could reinforce the adverse labour outcomes [40]. Moreover, it is possible that the discrepancy based on schooling is related to behavioural factors. Tendencies for health care seeking could, as for sex, differ between SES groups, which could explain the larger associations between CMD and labour outcomes among high-educated individuals.

CMD is associated with adverse labour outcomes in all age groups, although the relationships over age are not consistent. For instance, following a CMD diagnosis the youngest age group has significantly higher odds of non-employment and disability pension compared to older groups, but significantly lower odds of sick leave compared to 40–49-year olds. Worth noting regarding disability pension is that 20–29-year olds are covered by “activity compensation” instead of “sickness compensation” which is the case for the older groups. Although similar in many respects, activity compensation has a stronger focus on rehabilitation and covers individuals who need longer time to complete mandatory and secondary schooling due to disability and/or sickness. The results are also highly sensitive to the way we control for prior disability pension, by exclusion from the sample or as a control-variable. This is likely due to that there are relatively few new cases of disability pension (*n* = 287 out of which *n* = 248 were cases), but still requires that the results on disability should be interpreted with some caution.

Previous research on age differences in the association between CMD and labour outcomes found that adverse outcomes in employment status increased with age [21, 25] while sick leave due to CMD decreased with age, but only among women [30]. Conversely, we find that the adverse association between CMD and employment decrease with age. These results likely reflect a weaker labour force attachment among the youngest individuals. In Sweden, workers are often covered by comprehensive labour protection laws which are stronger the longer you have worked, and thus, often stronger the older you are. It is also possible that younger individuals have more options to paid employment such as continued studies or direct parental support. More research is needed to disentangle these observed variations over age.

Finally, our results show that labour outcomes following a CMD diagnosis differ also by country of birth. Individuals born in Sweden have higher odds of sick leave, while those born outside of Sweden have higher odds of non-employment, following a CMD diagnosis. When we exclude those who held disability pension the year before diagnosis it also appears as the odds of disability pension are significantly higher among the foreign-born. Similarly as for sex-, schooling- and age-related differences, these variations may be linked to health-related behaviour and attachment to the labour force.

As mentioned earlier, heterogeneity in the association between CMD diagnosis and labour outcomes across sex, age, SES and country of birth could impact on societal inequalities in labour outcomes, both through actual differences in the labour outcomes but also through further social stratification from disadvantageous consequences in health, see for example Diderichsen et al. (2012) [41]. This is the case for employment inequality based on country of birth. Predicted employment is lower among the foreign-born compared to those born in Sweden and a CMD diagnosis is associated with significantly lower employment among the foreign-born. As a result, the gap in employment increases between individuals born inside/outside Sweden. This is expected to be related also to increased income inequality and potentially increased health inequality given a negative correlation between unemployment and health.

Heterogeneity in the association between CMD diagnosis and the labour outcomes over population subgroups could also reduce already existing societal inequalities. For example, predicted sick leave is higher among women compared to men. However, the association between CMD diagnosis and sick leave is significantly higher among men. Consequently, heterogeneity in this association reduces the gap in sick leave between men and women, at least in relative terms. Similarly, the larger association between CMD diagnosis and non-employment among the highest educated also reduce employment inequalities between low and high educated individuals, but it is worth noting that employment rates still are significantly lower among the lower educated.

Heterogeneity in the association between CMD diagnosis and labour outcomes in combination with uneven disease onset and the complexity of different underlying causes between different population subgroups highlights the need of a system approach in future studies where the contribution of each step to societal inequalities is quantified. This is not to say that since, for example, women are on sick leave more often than men, we should not care that the association between CMD diagnosis and sick leave is larger among men. However, in order to mitigate the consequences of CMD among those worse off, it is crucial to know how labour outcomes following CMD are distributed between different population subgroups. Our results show that it can be motivated to focus inventions on particular groups if the policy purpose is both to reduce mental ill-health and to reduce societal inequalities.

### Strengths and weaknesses

A strength of this study is that it covers all individuals with a CMD diagnosis in one region in Sweden (Region Skåne). The control group, however, is drawn from a national representative sample. The population of Skåne is younger, higher educated, and more often foreign-born, compared to the population of Sweden in general [42]. Likely, there are other unobserved differences between these populations but matching on sex, age, schooling and country of birth adjust for a substantial part of the regional differences in population characteristics that exists. Still, it is possible that some variations remain, such as labour opportunities, which we cannot control for. For example, we know that Skåne has a higher concentration of foreign-born individuals. Labour market opportunities could be poorer if resources for support upon entry to the labour market are to be shared by many. Opportunities could also be better because a larger inflow of immigrants has forced the region to become more efficient regarding immigrants’ entry to the labour market. The high level of welfare and health care coverage in Sweden, and the fact that sick leave longer than 14 days and disability pension is handled by the Swedish Social Insurance Agency (national level) should mitigate regional differences in the labour outcomes, but the risk that such differences are reflected in our results is still worth noting.

Mental problems often occur early in life and diagnosed mental disorders are often preceded by less severe problems that may not have received clinical attention. Still, these problems may have had an effect on human capital accumulation and labour outcomes. There are significant differences between the case- and control groups in terms of labour outcomes the year before diagnosis. In all regressions, we control for the outcome variable the year before diagnosis since this has shown to be a strong predictor of labour outcomes. Likely though, the estimated associations do still to some extent reflect adverse labour outcomes prior to the diagnosis. In sensitivity analyses, we also exclude individuals with disability pension the year before diagnosis which substantially changes the odds of disability pension following a diagnosis. However, the main focus of this study is to establish heterogeneity in the relationships between a CMD diagnosis and labour outcomes, not to find causal estimates of CMD as such.

Another point worth raising is that a new treatment policy was introduced during the study period that meant that some of the cases received standard care and some received care that focused heavily on Cognitive Behaviour Therapy (CBT). The identification in this study is on the association between a CMD diagnosis and the labour outcomes and does not consider if the person gets treatment and if so what kind of treatment. However, we know that CBT more efficiently reduced adverse labour market outcomes compared to standard care and that higher educated individuals were more likely to be early receivers of the new policy and get CBT [43]. This could affect our results in two ways. First, the policy meant that more individuals got CBT compared to standard care, which would, given a higher effectiveness, lead to a reduced odds of non-employment following CMD diagnosis. Second, since those who got CBT (the more effective treatment) were more likely to be higher educated, it could affect heterogeneity based on education. The socioeconomic gradient in access/utilisation of health care is well-known and therefore we do not consider this as a cause for bias, but rather as a potential explanation of heterogeneity between population subgroups.

## Conclusion

The labour outcomes following a CMD diagnosis appear to differ based on sex, age, education, and country of birth. This heterogeneity sometimes contributes to and sometimes mitigates labour inequalities. When developing new strategies to tackle mental ill-health in the population, it may therefore be motivated to consider not only inequalities in the prevalence of mental disorders but also heterogeneity in associated adverse labour outcomes.

## Supplementary information

**Supplementary file 1: Table A1.** Logistic regression (odds ratios) – The association between CMD and subsequent labour outcomes.

## Abbreviations

CBT: Cognitive behavioural therapy
CMD: Common mental disorders
SES: Socioeconomic status
