# Supplementary material for: Heterogeneity in the associations between common mental disorders and labour outcomes – a population study from southern Sweden
Source: BMC Public Health. 2020 Aug 26;20:1285. doi: 10.1186/s12889-020-09348-3 (PMC7449029; doi:10.1186/s12889-020-09348-3)
Supplement: Supplementary file 1 — Table A1. Logistic regression (odds ratios) – The association between CMD and subsequent labour outcomes. [file 12889_2020_9348_MOESM1_ESM.docx]

Table A1 Logistic regression (odds ratios) – The association between CMD and subsequent labour outcomes

|  | Non-employed  Model 1  (1) | Non-employed  Model 2  (2) | Sick leave  Model 1  (3) | Sick leave  Model 2  (4) | Disability pension  Model 1  (5) | Disability pension  Model 2  (6) |
| --- | --- | --- | --- | --- | --- | --- |
| No CMD | Ref. | Ref. | Ref. | Ref. | Ref. | Ref. |
| CMD | 3.74  [3.58 - 3.91] | 3.92  [3.38 - 4.54] | 2.84  [2.74 - 2.94] | 1.79  [1.57 - 2.04] | 1.31  [1.16 - 1.47] | 2.20  [1.37 - 3.52] |
| Woman | Ref. | Ref. | Ref. | Ref. | Ref. | Ref. |
| Man | 1.15  [1.10 - 1.20] | 0.97  [0.90 - 1.05] | 0.55  [0.53 - 0.57] | 0.47  [0.45 - 0.50] | 1.23  [1.09 - 1.39] | 1.10  [0.89 - 1.35] |
| Mandatory education | Ref. | Ref. | Ref. | Ref. | Ref. | Ref. |
| Secondary education | 0.69  [0.65 - 0.73] | 0.72  [0.66 - 0.80] | 1.25  [1.18 - 1.31] | 1.01  [0.93 - 1.10] | 0.78  [0.68 - 0.89] | 0.78  [0.63 - 0.97] |
| Higher education | 0.48  [0.45 - 0.51] | 0.44  [0.39 - 0.49] | 0.98  [0.93 - 1.04] | 0.69  [0.64 - 0.76] | 0.57  [0.48 - 0.67] | 0.52  [0.39 - 0.68] |
| 20-29 years | Ref. | Ref. | Ref. | Ref. | Ref. | Ref. |
| 30-39 years | 0.43  [0.41 - 0.46] | 0.48  [0.43 - 0.53] | 1.68  [1.59 - 1.78] | 1.61  [1.48 - 1.76] | 0.69  [0.55 - 0.88] | 1.02  [0.67 - 1.56] |
| 40-49 years | 0.38  [0.36 - 0.40] | 0.42  [0.38 - 0.46] | 1.66  [1.57 - 1.76] | 1.54  [1.41 - 1.68] | 1.18  [0.94 - 1.47] | 1.84  [1.25 - 2.72] |
| 50-59 years | 0.59  [0.56 - 0.63] | 0.73  [0.66 - 0.80] | 1.61  [1.52 - 1.71] | 1.60  [1.46 - 1.74] | 3.37  [2.69 - 4.22] | 4.77  [3.24 - 7.02] |
| Born in Sweden | Ref. | Ref. | Ref. | Ref. | Ref. | Ref. |
| Foreign born | 1.61  [1.53 - 1.70] | 1.47  [1.35 - 1.60] | 0.88  [0.84 - 0.92] | 1.01  [0.95 - 1.09] | 1.08  [0.94 - 1.23] | 1.21  [0.97 - 1.51] |
| Outcome year -1 | 34.15  [32.06 - 36.38] | 34.61  [32.48 - 36.88] | 5.74  [5.48 - 6.02] | 5.73  [5.46 - 6.00] | 928  [809 - 1,066] | 926  [807 - 1,063] |
| Income Q1 | Ref. | Ref. | Ref. | Ref. | Ref. | Ref. |
| Income Q2 | 0.74  [0.69 - 0.78] | 0.74  [0.69 - 0.78] | 1.30  [1.23 - 1.37] | 1.30  [1.23 - 1.38] | 1.17  [0.99 - 1.37] | 1.17  [1.00 - 1.37] |
| Income Q3 | 0.61  [0.58 - 0.65] | 0.62  [0.58 - 0.66] | 1.59  [1.50 - 1.68] | 1.58  [1.50 - 1.67] | 1.20  [1.02 - 1.42] | 1.21  [1.02 - 1.43] |
| Income Q4 | 0.39  [0.37 - 0.42] | 0.39  [0.37 - 0.42] | 1.84  [1.74 - 1.94] | 1.83  [1.73 - 1.94] | 0.88  [0.73 - 1.07] | 0.89  [0.73 - 1.07] |
| Income Q5 | 0.28  [0.26 - 0.30] | 0.28  [0.26 - 0.31] | 1.59  [1.50 - 1.69] | 1.60  [1.51 - 1.69] | 0.69  [0.56 - 0.86] | 0.70  [0.57 - 0.87] |
| Constant | 0.58  [0.53 - 0.63] | 0.56  [0.50 - 0.64] | 0.10  [0.09 - 0.11] | 0.14  [0.12 - 0.15] | 0.00  [0.00 - 0.01] | 0.00  [0.00 - 0.00] |
| Man*CMD |  | 1.30  [1.19 - 1.43] |  | 1.26  [1.17 - 1.36] |  | 1.19  [0.93 - 1.54] |
| Secondary education*CMD |  | 0.92  [0.82 - 1.05] |  | 1.40  [1.26 - 1.55] |  | 0.99  [0.75 - 1.30] |
| Higher education*CMD |  | 1.15  [1.00 - 1.31] |  | 1.76  [1.58 - 1.97] |  | 1.15  [0.82 - 1.62] |
| 30-39 years*CMD |  | 0.85  [0.74 - 0.96] |  | 1.07  [0.96 - 1.20] |  | 0.56  [0.33 - 0.93] |
| 40-49 years*CMD |  | 0.85  [0.75 - 0.96] |  | 1.13  [1.01 - 1.26] |  | 0.51  [0.32 - 0.81] |
| 50-59 years*CMD |  | 0.71  [0.63 - 0.81] |  | 1.00  [0.90 - 1.12] |  | 0.59  [0.37 - 0.94] |
| Foreign born*CMD |  | 1.18  [1.05 - 1.31] |  | 0.79  [0.72 - 0.87] |  | 0.83  [0.63 - 1.09] |
|  |  |  |  |  |  |  |
| n | 76,608 | 76,608 | 76,608 | 76,608 | 76,608 | 76,608 |
| Pseudo-R^2^ | 0.39 | 0.39 | 0.14 | 0.14 | 0.78 | 0.78 |

Note: Non-employment in November at least one of the three follow-up years. Sick leave >14 days and disability pension at least once during the follow-up. The interaction effect shows the difference in associations between CMD diagnosis and the labour outcome for the interacted group compared to the reference group, as a ratio of odds ratios. The reference groups are women, mandatory education, 20–29 years of age and born in Sweden. We control for sex, education, age-group, being foreign-born, income and year of diagnosis/inclusion in the study in all regressions. Income is measured by disposable income in the year before diagnosis and is stratified in quintiles.
